# Quantitative videocapillaroscopy correlates with functional respiratory parameters: a clue for vasculopathy as a pathogenic mechanism for lung injury in systemic sclerosis

**DOI:** 10.1186/s13075-018-1775-9

**Published:** 2018-12-19

**Authors:** Alfredo Guillén-Del-Castillo, Carmen Pilar Simeón-Aznar, Eduardo L. Callejas-Moraga, Carles Tolosa-Vilella, Serafín Alonso-Vila, Vicente Fonollosa-Pla, Albert Selva-O’Callaghan

**Affiliations:** 1 Department of Systemic Autoimmune Diseases, Hospital Universitari Vall d’Hebron, Universitat Autònoma de Barcelona, Passeig Vall d’Hebron 119–129, 08035 Barcelona, Spain; 2 Department of Internal Medicine, Corporació Sanitària Universitària Parc Taulí, Universitat Autònoma de Barcelona, Sabadell, Barcelona, Spain

**Keywords:** Interstitial lung disease, Nailfold videocapillaroscopy, Pulmonary hypertension, Scleroderma, Systemic sclerosis

## Abstract

**Background:**

To determine whether lung involvement is related to microvascular perturbations, nailfold videocapillaroscopy (NVC) was performed in patients with systemic sclerosis (SSc).

**Methods:**

A cross-sectional study was consecutively accomplished in 152 SSc patients. NVC, a pulmonary function test and echocardiography were undergone within a 3-month period. Finally, 134 patients with at least eight NVC (200× magnification) images were selected for quantitative and qualitative examinations.

**Results:**

Patients with interstitial lung disease presented lower median capillary density (4.86/mm vs 5.88/mm, *p* = 0.005) and higher median of neoangiogenesis (0.56/mm vs 0.31/mm, *p* = 0.005). A higher quantity of neoangiogenesis capillaries was found in patients with pulmonary arterial hypertension (0.70/mm vs 0.33/mm, *p* = 0.008). Multivariate linear regression analysis established a correlation between neoangiogenesis and decreased forced vital capacity (FVC) (*p* < 0.001): for each capillary with neoangiogenesis visualized on average per 1 mm, FVC was 7.3% reduced. In qualitative NVC, a late pattern as defined by Cutolo was also associated with lower FVC (*p* = 0.018). The number of giant capillaries was associated with reduced diffusion capacity of the lung for carbon monoxide (DLCO) (*p* = 0.016); for each giant capillary per 1 mm, DLCO was 11.8% diminished.

**Conclusions:**

A good correlation was observed between distinctive quantitative and qualitative NVC features with lung functional parameters such as FVC and DLCO. It is suggested that vasculopathy could play a role in SSc lung involvement.

## Background

Systemic sclerosis (SSc) is a connective tissue disease characterized by microvascular damage. It seems that endothelial activation induces the release of inflammatory mediators, platelet activation and inflammatory cell recruitment [1–3]. Subsequently, intimal and smooth muscle vessel wall proliferation ensues, causing luminal obstruction and activation of myofibroblasts that results in architectural alterations due to an excessive extracellular matrix deposition, reduction in the number of capillaries, tissue hypoxia and dysfunction of vascular regeneration [4].

The lung is one of the most frequently jeopardized organs in SSc; thus interstitial lung disease (ILD) affects around 55–65% of patients subjected to high-resolution computed tomography (HRCT) [5, 6], and pulmonary arterial hypertension (PAH) diagnosed by right heart catheterization (RHC) involves approximately 10% of this population [7, 8]. As a result of its high prevalence and poor prognosis, pulmonary involvement is nowadays the major cause of SSc-related deaths [9–11]. An exhaustive study of the relation between capillaroscopy features and lung manifestations would facilitate comprehension of the pulmonary pathophysiology.

Capillary microscopy was initially used as a qualitative technique to describe microvascular abnormalities in SSc and other autoimmune-related diseases. Maricq et al. [12] defined the scleroderma pattern as a group of capillaroscopy findings with potential diagnostic value in this disease. Later, these were detailed into active and slow capillaroscopy patterns [13]. More recently, using nailfold videocapillaroscopy (NVC), Cutolo et al. [14] defined a new classification of qualitative capillaroscopy abnormalities into early, active and late scleroderma patterns. The late pattern was related to lower levels of circulating endothelial progenitor cells and higher vascular endothelial growth factor (VEGF), demonstrating that an impairment of vasculogenesis mediates the loss of capillaries [15]. Recently, a sequential quantitative NVC study relating the presence of new incident capillaroscopy features to disease progression has been reported [16]. Nevertheless, no prior studies were focused on extensive quantitative NVC analysis and its correlation with objective lung parameters.

The hypothesis of the present study was that microcirculation damage leads the pathogenesis of the main manifestations in SSc such as ILD and PAH. The main aim of the work was to analyse NVC alterations using a quantitative method and to investigate their correlation with the pulmonary function test (PFT).

## Methods

### Patients

A cross-sectional study was carried out on SSc patients from the Vall d’Hebron Hospital cohort. All patients fulfilled LeRoy et al.’s SSc classification criteria [17], and accomplishment of the 2013 ACR/EULAR classification criteria was also assessed [18]. The study was approved by the Ethics Committee for Clinical Research (PG(AG)4/2015), and all patients provided written informed consent for their participation. NVC was consecutively conducted in 152 patients over a 12-month period. PFTs and echocardiography were performed within a 3-month period. Thirteen patients were excluded with fewer than eight explored nailfold fields, and another five patients for being lung transplant recipients. Finally, 134 SSc patients were selected for the analyses. The disease onset was described as the date of first symptom attributable to SSc including Raynaud’s phenomenon (RP). All clinical or parameter data were collected at the time of NVC. Study quality was assessed by the Strengthening the Reporting of Observational Studies in Epidemiology (STROBE) checklist for cross-sectional studies [19].

### Clinical manifestations

Cutaneous subsets were defined as previously [20], according to the extent of skin thickening: limited cutaneous SSc (lcSSc), if skin sclerosis was distal to the elbows and knees or the face; diffuse cutaneous SSc (dcSSc), if skin sclerosis was extended proximally to the elbows or knees; and sine scleroderma SSc (ssSSc), if there was no skin involvement. Manifestations collected included RP, telangiectasias, past or current digital ulcers (DU), calcinosis and past history of scleroderma renal crisis (SRC).

Interstitial lung disease was defined as radiologic evidence of interstitial findings on HRCT examined by an expert thorax radiologist [21]. PAH was defined as mean pulmonary arterial pressure (mPAP) ≥ 25 mmHg with pulmonary artery wedge pressure (PAWP) ≤ 15 mmHg, and pulmonary vascular resistance (PVR) > 3 Wood units in right heart catheterization (RHC) [22, 23]. Cardiac involvement was defined as past or current pericardial effusion, left ventricular ejection fraction (LVEF) < 50%, macrovascular or microvascular ischemic heart disease with no cardiovascular risk factor (CVRF), conduction abnormalities, diastolic dysfunction with no CVRF or mitral regurgitation with no CVRF [24].

Musculoskeletal disease included arthritis, tendon fiction rubs, joint contractures and myositis. Gastrointestinal SSc disease was established if any of the following were present: oesophageal dysmotility, gastric antral vascular ectasia, gastric or bowel dysmotility, intestinal bacterial overgrowth, intestinal pseudo-obstruction or anal sphincter dysfunction.

### Immunology features

Antinuclear antibodies (ANA) were evaluated by indirect immunofluorescence (IIF) assay using HEp-cell line 2. Anticentromere antibodies (ACA) were described by IIF, and anti-topoisomerase I antibodies were determined by enzyme-linked immunosorbent assay. A commercial line blot assay was performed to detect anticentromere proteins A and B, anti-RNA pol III and anti-PM/Scl antibodies (EUROLINE Systemic Sclerosis (Nucleoli) Profile (IgG), Euroimmun, Germany) according to the manufacturer’s instructions.

### Pulmonary function test and echocardiography

Complete PFTs including the percentage of the predicted forced vital capacity (FVC) and the percentage of the predicted diffusion capacity of the lung for carbon monoxide (DLCO) were accomplished using MasterLab equipment (MasterLab, Jaegger, Germany), following the ATS/ERS recommendations [25]. Echocardiography was performed by an experienced echocardiographer using a Vivid E9 system (General Electric Vingmed, Horten, Norway), according to the consensus guidelines [26]. Right ventricular systolic pressure (RVSP), tricuspid regurgitation velocity (TRV) and the presence of other echo pulmonary hypertension (PH) signs were measured. In order to avoid loss of data information about patients with no measurable TRV, the variable ‘TRV ≥ 2.9 m/s or other echo PH signs’ was created [23].

### Nailfold videocapillaroscopy

Nailfolds were examined using an Optilia Digital Videocapillaroscope (Optilia Instruments AB, Sollentuna, Sweden) with 200× lens magnification and an LED lamp by the same operator (AS-O) who was blinded to clinical patients’ conditions. Each patient was acclimatized for at least 20 min at room temperature of 20–24 °C before the examination. NVC was conducted with a contact adapter and immersion oil dropper, studying the middle nailfold from the second to fifth fingers of both hands. Optipix Lite software (Optilia Instruments AB) was used for visualization of consecutive 1-mm-wide images of first-line capillaries. Two images were taken on each finger, although when a finger was not possible to evaluate additional pictures were taken on other fingers. Patients with fewer than eight images were excluded from the study. Quantitative and qualitative analyses of the images were performed blind to clinical data by investigator AS-O. Inter-observer reliability and intra-observer reliability were also explored with a calculated sample size of 45 patients. This sample was randomly selected and blindly assessed once by investigator AG-DC to calculate the inter-observer reproducibility. Afterwards, to estimate the intra-observer repeatability, a second round was independently performed by investigators AS-O and AG-DC with a minimal time interval of 2 weeks between both measurements.

**Fig. 1:**
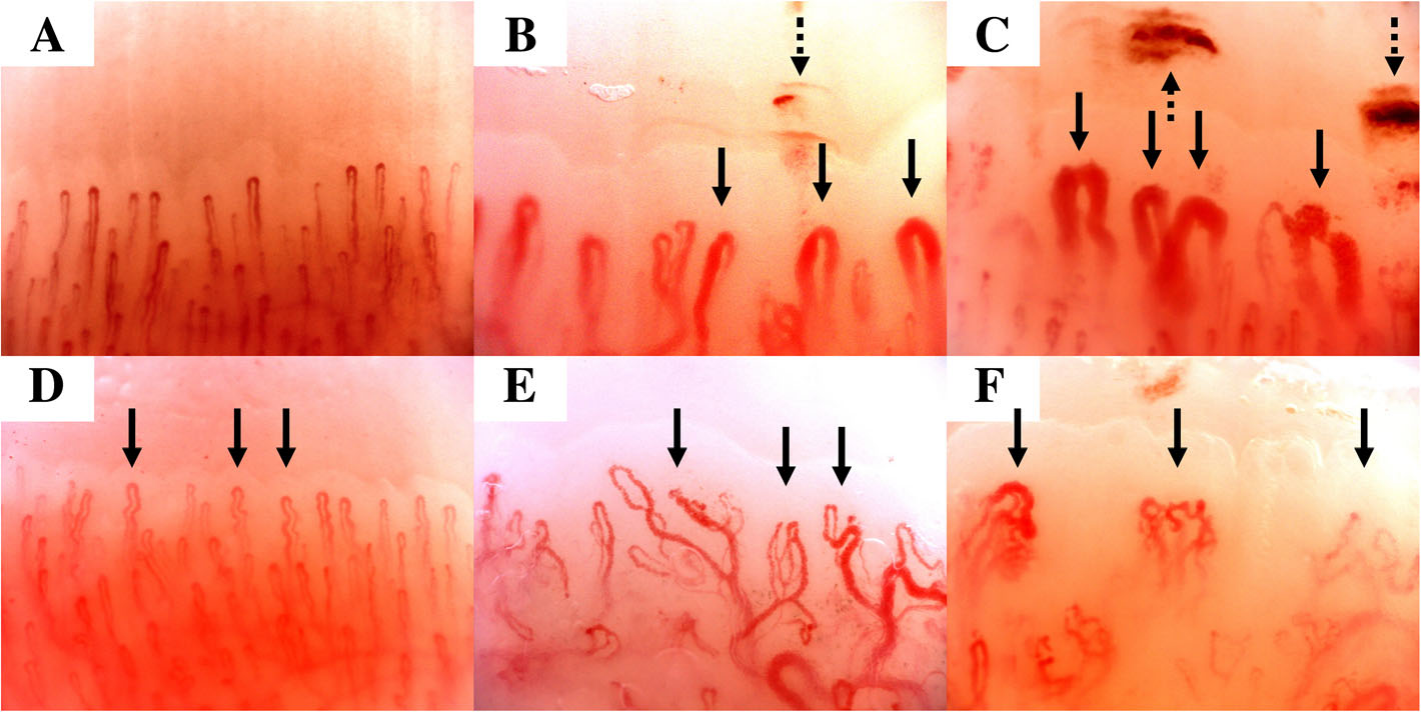
Quantitative nailfold videocapillaroscopy: **a** normal capillaries; **b** enlarged capillaries (solid arrows) and microhaemorrhage (dashed arrow); **c** giant capillaries (solid arrows) and microhaemorrhages (dashed arrows); **d** tortuous capillaries (solid arrows); **e** capillaries with neoangiogenesis as ramified capillaries (solid arrows); **f** capillaries with neoangiogenesis as disorganized capillaries (solid arrows)

Quantitative NVC was carried out according to previous definitions as follows: capillary density, number of capillaries measured in the distal row following the 90° method [27]; enlarged capillary, an increase in capillary diameter > 20 μm; giant capillary, a capillary dilatation > 50 μm; microhaemorrhage, distal haemosiderin deposits; tortuous, curled capillaries with no cross; and neoangiogenesis, branching, bizarre, bushy, disorganized, ramified or arborized capillaries (Fig. 1) [14, 28, 29].

The mean of each capillaroscopic feature was calculated from the sum of consecutive images for each digit. Subsequently, the average values from eight fingers were added together and divided by the number of studied digits. The resulting value indicated the number of this capillaroscopic feature adjusted by each millimetre of the nailfold.

Qualitative NVC analysis of images was performed following capillaroscopy patterns described by Cutolo et al. (normal, early, active and late) [14].

### Statistical analysis

For the descriptive statistics, qualitative data were expressed as the mean and standard deviation (SD) after approving the normal distribution test; and non-normal qualitative variables were described as the median and interquartile range (IQR). Normal distribution was assessed using the Kolmogorov–Smirnov test. To assess whether there were statistically significant differences between groups of patients, the Mann–Whitney *U* test was used. Bonferroni correction was applied in multiple comparisons. Associations between NVC and clinical features, PFTs and echocardiography variables were analysed using Spearman’s correlation.

In order to analyse inter-observer and intra-observer reliability, a sample size of 45 patients was calculated, based on two observers (AS-O and AG-DC), with a power of 80% to detect that an intra-class coefficient (ICC) of 0.7 was significantly higher than 0.45 [30, 31]. The value of 0.45 was arbitrarily selected as the lowest acceptable limit of reliability. Quantitative NVC features were evaluated as the number per millimetre and determined by the ICC, whereas qualitative NVC features were assessed by Cohen’s kappa coefficient. The measurement of observer agreement was defined as described in the literature: < 0.00 = poor; 0.00–0.20 = slight; 0.21–0.40 = fair; 0.41–0.60 = moderate; 0.61–0.80 = substantial; and 0.81–1.00 = almost perfect [32].

Multiple linear regression tests were performed to determine the association with FVC and DLCO. The multivariate models for FVC included NVC features, age at NVC, time elapsed from first symptom to NVC, dcSSc subset, gender, interstitial findings on HRCT, different autoantibodies and DU. For DLCO models, FVC, PAH confirmed by RHC, TRV ≥ 2.9 m/s or other echo PH signs also were introduced. *p* < 0.05 was considered significant. Statistical analysis was conducted with SPSS 20.0 for Windows (SPSS Inc., Chicago, IL, USA).

## Results

### Population characteristics

Demographic characteristics of the 134 SSc patients are presented in Table 1. The participants were mainly female (*n* = 113, 84.3%) and in the lcSSc subset (*n* = 88, 65.7%). More than 90% of the population fulfilled the 2013 ACR/EULAR classification criteria. ACA were the most common antibodies in 49 (36.6%) patients, followed by anti-topoisomerase I antibodies in 31 (23.1%) patients. Considering major organ involvement, digestive involvement was present in 110 (82.1%) patients, cardiac disease in 103 (76.9%) patients, ILD in 58 (43.3%) patients, musculoskeletal involvement in 40 (29.9%) patients and PAH in 11 (8.2%) patients.

**Table 1:** Demographic, clinical and immunological characteristics of the patients with SSc (*n* = 134, 100%)

Variable	Result
Age at SSc onset (years)	38.5 (± 15.7)
Age at NVC (years)	54.7 (± 14.4)
Cutaneous subsets, dcSSc/lcSSc/ssSSc	28 (20.9)/88 (65.7)/18 (13.4)
Time elapsed from first symptom to NVC (years)	16.0 (± 12.6)
Time elapsed from first non-RP symptom to NVC (years)	10.9 (± 9.9)
2013 ACR/EULAR SSc classification criteria fulfilment	121 (90.2)
ACA/ATA/anti-RNA pol III/anti-PM/Scl	49 (36.6)/31 (23.1)/14 (10.4)/11 (8.2)
Raynaud’s phenomenon	132 (98.5)
Telangiectasias	97 (72.4)
Digital ulcers	62 (46.3)
Calcinosis	29 (21.6)
Scleroderma renal crisis	2 (1.5)
Interstitial lung disease	58 (43.3)
Pulmonary arterial hypertension	11 (8.2)
Cardiac involvement	103 (76.9)
Musculoskeletal disease	40 (29.9)
Gastrointestinal involvement	110 (82.1)
Overlap features	19 (14.1)

Data presented as mean (± standard deviation) for continuous variables and as number (%) for categorical variables

*SSc* systemic sclerosis, *NVC* nailfold videocapillaroscopy, *dcSSc* diffuse cutaneous systemic sclerosis, *lcSSc* limited cutaneous systemic sclerosis, *ssSSc* sine scleroderma systemic sclerosis, *RP* Raynaud’s phenomenon, *ACR* American College of Rheumatology, *EULAR* European League Against Rheumatism, *ACA* anticentromere antibodies, *ATA* anti-topoisomerase I

The distribution of different cardiac manifestations was 76.9% of patients presenting cardiac conduction abnormalities, 57.5% mitral regurgitation (with no CVRF), 47% diastolic dysfunction (with no CVRF), 13.4% pericardial effusion, 8.2% microvascular ischemic heart disease (with no CVRF), 3.7% LVEF < 50% and 3% of patients macrovascular ischemic heart disease (with no CVRF). Among all patients, there were 19 (14.1%) with overlap features as follows: nine (6.7%) patients had dermatomyositis, seven (5.2%) patients had Sjögren syndrome and the other three (2.2%) patients had rheumatoid arthritis.

### Pulmonary assessments

PFTs and echocardiography findings are presented in Table 2. The mean predicted FVC (± SD) was 80.8% (± 20.1), with a mean predicted DLCO of 66.2% (± 23.7). Regarding echocardiography, the TRV could be measured in 96 patients with a mean of 2.8 m/s (± 0.3), and no other signs of PH were found in the remaining 38 patients. Among 14 (10.4%) subjects with TRV higher than 2.9 m/s and/or with other echo PH signs, seven patients had been previously diagnosed with PAH by RHC. On the other hand, only 4 out of 120 patients with TRV lower than 2.9 m/s and no other echo PH signs had been formerly diagnosed with PAH by RHC.

**Table 2:** Pulmonary function test and echocardiography within a 3-month period of nailfold videocapillaroscopy

Variable	*N*	Result
Pulmonary function test		
FVC, % predicted	134	80.8 (± 20.1)
DLCO, % predicted	134	66.2 (± 23.7)
FVC%/DLCO%	134	1.5 (± 0.4)
Echocardiography		
RVSP (mmHg)	96	30.7 (± 12.0)
TRV (m/s)	96	2.8 (± 0.3)
TRV ≥ 2.9 m/s or other echo PH signs	134	14 (10.4)

Data presented as mean (± standard deviation) for continuous variables and as number (%) for categorical variables

*FVC* forced vital capacity, *DLCO* diffusion capacity of the lung for carbon monoxide, *RVSP* right ventricular systolic pressure, *TRV* tricuspid regurgitation velocity, *PH* pulmonary hypertension

### NVC characteristics

A total of 2186 images were analysed from 134 patients, with a mean of 16.3 (± 5.0) images per subject and the median (IQR) of images for each finger was 2 (1–3). The median (IQR) of capillary density adjusted by each millimetre of nailfold was 5.44/mm (4.33–6.74) (Table 3). NVC findings according to the presence of ILD were compared. Median capillary density in ILD patients was 4.86/mm, significantly lower than patients with no ILD whose density was 5.88/mm (*p* = 0.005). Moreover, the number of capillaries with neoangiogenesis was higher in ILD patients (0.56/mm vs 0.31/mm, *p* = 0.005). Focusing on PAH, patients with this manifestation also showed greater frequency of capillaries with neoangiogenesis compared with patients with no PAH (0.70/mm vs 0.33/mm, *p* = 0.007). All prior comparisons remained statistically significant after Bonferroni correction (*p* < 0.008).

**Table 3:** Nailfold videocapillaroscopy characteristics and comparisons between groups of patients depending on pulmonary involvement

	ILD comparisons		*p* value	PAH comparisons		*p* value
	ILD (*n* = 58)	No ILD (*n* = 76)		PAH (*n* = 11)	No PAH (*n* = 123)	
Quantitative NVC features						
Capillary density (*n*) ª	4.86 (4.14–5.80)	5.88 (4.74–6.85)	0.005*	5.47 (5.00–5.83)	5.42 (4.31–6.74)	0.997
Enlarged capillaries (*n*) ª	0.84 (0.31–1.53)	0.85 (0.39–1.58)	0.738	0.56 (0.25–0.93)	0.91 (0.40–1.61)	0.128
Giant capillaries (*n*) ª	0.26 (0.00–0.64)	0.30 (0.04–1.07)	0.362	0.06 (0.00–0.40)	0.29 (0.03–1.02)	0.167
Microhaemorrhages (*n*) ª	0.12 (0.00–0.38)	0.13 (0.00–0.42)	0.735	0.06 (0.00–0.22)	0.14 (0.00–0.40)	0.101
Tortuous capillaries (*n*) ª	0.45 (0.19–1.31)	0.72 (0.28–1.49)	0.216	0.63 (0.25–1.71)	0.59 (0.23–1.43)	0.773
Neoangiogenesis (*n*) ª	0.56 (0.23–1.28)	0.31 (0.12–0.62)	0.005*	0.70 (0.47–1.80)	0.33 (0.12–0.78)	0.007*
Qualitative NVC features						
Cutolo’s pattern, early/active/late	8 (13.8)/23 (39.7)/23 (39.7)	15 (19.7)/36 (47.4)/14 (18.4)	0.366/0.373/0.006*	1 (9.1)/4 (36.4)/6 (54.5)	21 (17.9)/52 (44.4)/29 (24.8)	0.688/0.755/0.069

Data presented as median (interquartile range) for continuous variables and as number (%) for categorical variables

*ILD* interstitial lung disease, *PAH* pulmonary arterial hypertension, *NVC* nailfold videocapillaroscopy

ªAdjusted by each millimetre of the nailfold

*Statistically significant comparison after Bonferroni correction (*p* < 0.008)

Concerning qualitative NVC analysis, a late pattern (27.6%) was more prevalent in patients with ILD (*p* = 0.006). In addition, a late pattern showed lower FVC and DLCO values compared with normal, early and active patterns (Table 4).

**Table 4:** Association between qualitative nailfold videocapillaroscopy and pulmonary function tests

	Cutolo’s pattern				
	Normal (*n* = 15)	Early (*n* = 23)	Active (*n* = 59)	Late (*n* = 37)	*p* value
FVC, % predicted	89.1 (± 20.3)	82.3 (± 17.2)	85.3 (± 18.3)	69.6 (± 20.7)*^,^**	0.001
DLCO, % predicted	77.3 (± 20.8)	71.4 (± 27.5)	68.6 (± 20.4)	54.2 (± 23.3)*^,†,‡^	0.002

Data presented as mean (± standard deviation) for continuous variables

*FVC* forced vital capacity, *DLCO* diffusion capacity of the lung for carbon monoxide

*p < 0.01, late versus normal

**p = 0.001, late versus active

^†^*p* < 0.05, late versus early

^‡^*p* < 0.05, late versus active

### Clinical and NVC correlations

Age at NVC was inversely correlated with the number of enlarged capillaries (*ρ* = − 0.20, *p* = 0.01) and the quantity of microhaemorrhages (*ρ* = − 0.25, *p* = 0.003). The time elapsed from first symptom to NVC was negatively associated with the number of microhaemorrhages (*ρ* = − 0.25, *p* = 0.003) and positively with the number of capillaries with neoangiogenesis (*ρ* = 0.18, *p* = 0.03). Higher capillary density was positively correlated with the % DLCO (*ρ* = 0.26, *p* = 0.003) and negatively with the RVSP (*ρ* = − 0.21, *p* = 0.03). The number of capillaries with neoangiogenesis was inversely associated with % FVC (*ρ* = − 0.24, *p* = 0.004) and % DLCO (*ρ* = − 0.26, *p* = 0.002) and was positively associated with RVSP (*ρ* = 0.20, *p* = 0.04).

### Inter-observer and intra-observer reliability

The inter-observer reproducibility for all quantitative NVC features was almost perfect with an ICC higher than 0.80 (Table 5), except for the number of capillaries with neoangiogenesis which displayed a substantial reproducibility (95% CI) of 0.77 (0.58–0.88). Qualitative Cutolo’s patterns also showed almost perfect reproducibility with Cohen’s *κ* of 0.83 (0.66–1.0), even when each pattern was analysed separately, with the only exception of an early pattern which had a substantial reproducibility with Cohen’s *κ* of 0.73 (0.42–1.0).

**Table 5:** Inter-observer and intra-observer reliability

	Inter-observer (95% CI)	Intra-observer (95% CI)
ICC for quantitative NVC features		
Capillary density	0.96 (0.93–0.98)	0.97 (0.93–0.98)
Enlarged capillaries	0.87 (0.77–0.93)	0.93 (0.83–0.97)
Giant capillaries	0.91 (0.83–0.95)	0.95 (0.90–0.97)
Microhaemorrhages	0.97 (0.94–0.98)	0.99 (0.98–0.99)
Tortuous capillaries	0.92 (0.85–0.95)	0.91 (0.69–0.96)
Neoangiogenesis	0.77 (0.58–0.88)	0.81 (0.56–0.90)
Cohen’s *κ* coefficient for qualitative NVC features		
Cutolo’s patterns	0.83 (0.66–1.0)	0.81 (0.64–0.97)
Normal pattern	0.92 (0.75–1.0)	0.83 (0.55–1.0)
Early pattern	0.73 (0.42–1.0)	0.66 (0.33–0.99)
Active pattern	0.82 (0.65–0.99)	0.87 (0.71–1.0)
Late pattern	0.87 (0.69–1.0)	0.81 (0.59–10)

*CI* confidence interval, *ICC* intra-class coefficient, *NVC* nailfold videocapillaroscopy

Regarding intra-observer repeatability, similar results were obtained for quantitative and qualitative variables, with an increment of neoangiogenesis repeatability up to 0.81 (0.56–0.90).

#### Multivariate analysis

##### Quantitative NVC studies

To elucidate which NVC findings were more strongly associated with pulmonary parameters, a multivariate analysis was conducted using % FVC as a dependent variable. Table 6 presents variables with statistical significance or a tendency to be significant. The number of capillaries with neoangiogenesis in NVC, male gender and ILD on HRCT were associated with a lower % FVC, whereas ACA positivity was identified as a protective factor. The analysis estimated that for each capillary with neoangiogenesis visualized on average per 1-mm nailfold, the FVC would be reduced 7.3%.

**Table 6:** Multiple regression analysis according to quantitative nailfold videocapillaroscopy

	*B*	95% CI	*p* value
FVC as dependent variable			
Male gender	−11.0	−19.2 to −2.9	0.008
Interstitial findings on HRCT	−14.8	−21.1 to −8.4	< 0.001
ACA positivity	7.2	0.1 to 14.3	0.045
Neoangiogenesis/mm	−7.3	−11.0 to −3.6	< 0.001
DLCO as dependent variable			
Digital ulcers	−6.7	−13.6 to 0.1	0.056
Interstitial findings on HRCT	−9.4	−16.4 to −2.5	0.008
FVC	0.5	0.3 to 0.7	<0.001
PAH confirmed by RHC	−14.3	−26.9 to −1.6	0.027
TRV ≥ 2.9 m/s or other echo PH signs	−16.4	− 28.3 to −4.6	0.007
Enlarged capillaries	7.8	1.1 to 14.4	0.021
Giant capillaries	−11.8	−21.4 to −2.2	0.016

Only variables with statistical significance or tendency to be significant are presented

B regression coefficient, *CI* confidence interval, *FVC* forced vital capacity, *HRCT* high resolution computed tomography, *ACA* anticentromere antibodies, *DLCO* diffusion capacity of the lung for carbon monoxide, *PAH* pulmonary arterial hypertension, *RHC* right heart catheterization, *TRV* tricuspid regurgitation velocity, *PH* pulmonary hypertension

A lower % DLCO value was associated with a reduced number of enlarged capillaries and an increased number of giant capillaries, along with ILD on HRCT, lower % FVC, PAH confirmed by RHC, TRV ≥ 2.9 m/s or other echo PH signs on echocardiography. In this way, for each enlarged capillary on average per 1 mm the DLCO would be 7.8% higher; however, for each giant capillary it would be diminished 11.8%.

##### Qualitative NVC studies

Multivariate analysis showed an association between late pattern and lower % FVC values. FVC would be diminished 14.3% in a patient with late NVC pattern features (Table 7). None of the NVC patterns were related to DLCO values.

**Table 7:** Multiple regression analysis according to Cutolo’s patterns

	*B*	95% CI	*p* value
FVC as dependent variable			
Male gender	−12.3	−21.0 to −3.5	0.006
Interstitial findings on HRCT	−13.5	−20.2 to −6.7	< 0.001
ACA positivity	8.6	−0.1 to 17.4	0.051
Cutolo’s late pattern	−14.3	−26.1 to −2.5	0.018
DLCO as dependent variable			
Digital ulcers	−8.3	−14.8 to −1.8	0.013
Interstitial findings on HRCT	−9.8	−16.8 to −2.8	0.006
FVC	0.5	0.3 to 0.7	< 0.001
PH confirmed by RHC	−14.1	−26.8 to −1.5	0.028
TRV ≥ 2.9 m/s or other echo PH signs	− 14.9	−26.8 to −3.0	0.014

Only variables with statistical significance or tendency to be significant are presented

B regression coefficient, *CI* confidence interval, *FVC* forced vital capacity, *HRCT* high resolution computed tomography, *ACA* anticentromere antibodies, *DLCO* diffusion capacity of the lung for carbon monoxide, *PAH* pulmonary arterial hypertension, *PH* pulmonary hypertension, *RHC* right heart catheterization, *TRV* tricuspid regurgitation velocity

## Discussion

This cross-sectional study in an SSc population indicated that the presence of capillaries with neoangiogenesis in NVC was related to lower percentage FVC values independently of other baseline characteristics. According to our results, a patient with one capillary with neoangiogenesis per millimetre on average in NVC would have 7.3% lower FVC percentage regardless of other clinical or capillaroscopic conditions. Concerning qualitative NVC assessments, a late pattern was also associated with lower FVC. We also found a substantial or almost perfect inter-observer and intra-observer reliability of all NVC features.

The median capillary density was similar to that reported previously, but our study included a higher significant number of evaluated images per patient [33, 34]. Prior publications have mainly focused on qualitative NVC, finding correlations between NVC patterns and different variables such as ILD, FVC, DLCO, RVSP, DU or even risk of developing new DU [35–38]. On the other hand, quantitative NVC studies proposed capillaroscopic prognostic tools for the development of new DU or even the progression to SSc [34, 39–41]. We achieved equal or superior intra-observer and inter-observer reliability to previous quantitative NVC studies [27], probably because our work compared the mean of NVC features analysing all images of each patient instead of a single image per patient, which reflects real-life capillaroscopy more. Moreover, qualitative NVC features as Cutolo’s patterns were assessed with almost perfect reliability. However, few research projects have concentrated on extensive quantitative analysis and its correlation with clinical variables, specifically with lung parameters.

We found that SSc patients with ILD had a lower capillary density and a higher number of capillaries with neoangiogenesis. These findings support the prior semi-quantitative NVC study, which identified higher mean avascular scores in patients with ground-glass opacities [42]. Similarly, Castellvi et al. [43] described an association between low capillary density defined as < 7 capillaries/mm and lower FVC and DLCO percentages using a semi-quantitative NVC. Smith et al. [37] also found a strong association between NVC patterns and future severe lung involvement at 18–24 months, defined as a punctuation ≥ 2 in lung evaluation on the Medsger severity scale. Nevertheless, the risk of severe ILD or PH was not specified separately [37, 44].

Recently, Avouac et al. [16] published a sequential quantitative NVC study with multivariate Cox analysis revealing that an incident or increased number of giant capillaries during the follow-up was protective of new DU. Progressive loss of capillaries from baseline predicted overall disease progression, new DU, lung vascular progression defined as new onset of precapillary PH on RHC, skin fibrosis and worsening in the Medsger severity score. Baseline angiogenesis predicted only lung vascular progression. The present study performs a multivariate analysis including clinical, immunological and quantitative/qualitative NVC findings for the investigation of lung parameters at baseline conditions. We identified that the number of capillaries with neoangiogenesis, male gender and the presence of ILD on HRCT were factors independently associated with lower FVC values, while ACA positivity was found to be a protector, which supports Avouac et al.’s work.

Furthermore, regarding qualitative NVC studies, the progression from normal/early/active to a late pattern was related to the development of new DU, lung vascular progression, progression of skin fibrosis and worsening on the Medsger severity scale [16]. According to these findings, the greatest destructive NVC features, as the late pattern, were associated with lower FVC values.

Consequently, it appears that endothelial damage mediates both lung fibrosis and peripheral microvascular changes visualized in NVC. SSc patients with more extensive ILD may also show higher nailfold capillary perturbations, ineffective angiogenesis and vasculogenesis, which reflects a higher frequency of bushy or bizarre capillaries which are denominated as neoangiogenesis [45]. In fact, ILD has already been linked in a multivariate analysis with peripheral vasculopathy in the form of prior/current DU [20].

Recently, a qualitative NVC study has noted the predictive role of NVC and specific autoantibodies in cardiopulmonary involvement [35]. Specifically, active and late NVC patterns were associated with ILD, DLCO < 70% of predicted, reduced maximum oxygen uptake, higher NT pro-BNP and increased systolic pulmonary artery pressure values. However, PH was estimated by indirect measurements and was not confirmed by RHC. In our cohort, the prevalence of PAH confirmed by RHC was 8.2%, similar to previous reports [7, 8]. Surprisingly, we found enlarged capillaries being associated with higher % DLCO values, and giant capillaries were independently correlated with lower DLCO percentage. These findings contrast with a prior publication where new giant or increased giant capillaries during follow-up tended to be protective of lung vascular progression or overall disease progression [16]. This paradoxical association between enlarged capillaries and higher DLCO values could be explained as an adaptive phenomenon. When enlarged capillaries turn into giant capillaries, which means a failure in microvascular adaptation and consequently a severe capillary dysfunction, an impairment of DLCO values occurs. The formation of extremely large capillaries may contribute to gas exchange disturbance in all tissues including the lung, which might result in DLCO decline.

Hofstee et al. [46] found that capillary density was inversely correlated with the mPAP in both SSc-PAH and idiopathic PAH patients. Interestingly, Riccieri et al. [47] demonstrated the existence of greater avascular areas and severe active/late NVC patterns in a group of 12 SSc-PAH patients, and that higher NVC scores and avascular areas scores were correlated with mPAP. Corrado et al. [48] observed capillaroscopic abnormalities in 38.1% of idiopathic PAH patients, with lower capillary density and higher loop width compared to healthy subjects. Furthermore, the authors confirmed a reduced capillary density, and an increased mean capillary width and mean number of capillaries with neoangiogenesis in SSc-PAH patients compared to SSc patients without PAH evidence. Although we also identified a negative correlation of higher capillary density and number of neoangiogenesis capillaries with RVSP values, we did not found an association between those NVC features and PAH defined by RHC in the multivariate analysis, which may be due to the small group of PAH patients.

Limitations of this study included the cross-sectional study, with no follow-up data, which did not allow us to infer whether capillaroscopic findings were linked to a specific outcome of pulmonary disease. However, although an association was observed between microvascular features and functional respiratory parameters, this fact does not prove a causal relationship between them. We cannot exclude that both phenomena would be influenced by an unknown parameter that may lead a parallel change in the first two. Notwithstanding, the present study also had strengths, because all patients had undergone echocardiography and PFTs close to the NVC examination. The images were analysed blind of clinical data by the researchers. For the statistical analysis, patients were excluded with fewer than eight explored fields, either due to technical difficulties or digital amputations. NVC explorations after autologous stem cell transplantation had demonstrated an improvement in vascular damage [49]. Consequently, five lung transplant patients under high immunosuppressive treatment were additionally removed from the study.

## Conclusions

This study describes a lower capillary density and a higher number of capillaries with neoangiogenesis in patients with ILD, and demonstrates a quantitative and qualitative relation between specific NVC abnormalities and lung function tests (both % FVC and % DLCO parameters). It seems that NVC findings are linked to a wide spectrum of clinical variables in SSc, which emphasizes the crucial role of microcirculation damage in this medical condition, especially within the lung.

## Abbreviations

ACA: Anticentromere antibodies
ANA: Antinuclear antibodies
CI: Confidence interval
CVRF: Cardiovascular risk factor
dcSSc: Diffuse cutaneous SSc
DLCO: Diffusion capacity of the lung for carbon monoxide
DU: Digital ulcers
FVC: Forced vital capacity
HRCT: High-resolution computed tomography
ICC: Intra-class coefficient
IIF: Indirect immunofluorescence
ILD: Interstitial lung disease
IQR: Interquartile range
lcSSc: Limited cutaneous SSc
LVEF: Left ventricular ejection fraction
mPAP: Mean pulmonary arterial pressure
NVC: Nailfold videocapillaroscopy
PAH: Pulmonary arterial hypertension
PAWP: Pulmonary artery wedge pressure
PFT: Pulmonary function test
PH: Pulmonary hypertension
PVR: Pulmonary vascular resistance
RHC: Right heart catheterization
RP: Raynaud’s phenomenon
RVSP: Right ventricular systolic pressure
SD: Standard deviation
SRC: Scleroderma renal crisis
SSc: Systemic sclerosis
ssSSc: Sine scleroderma SSc
STROBE: Strengthening the Reporting of Observational Studies in Epidemiology
TRV: Tricuspid regurgitation velocity
VEGF: Vascular endothelial growth factor

## References

[1] Denton CP, Ong VH (2013). Targeted therapies for systemic sclerosis. Nat Rev Rheumatol.

[2] Allanore Y, Simms R, Distler O, Trojanowska M, Pope J, Denton CP (2015). Systemic sclerosis. Nat Rev Dis Primers.

[3] Denton CP, Khanna D (2017). Systemic sclerosis. Lancet.

[4] Bhattacharyya S, Wei J, Varga J (2011). Understanding fibrosis in systemic sclerosis: shifting paradigms, emerging opportunities. Nat Rev Rheumatol.

[5] Launay D, Remy-Jardin M, Michon-Pasturel U, Mastora I, Hachulla E, Lambert M (2006). High resolution computed tomography in fibrosing alveolitis associated with systemic sclerosis. J Rheumatol.

[6] De Santis M, Bosello S, La Torre G, Capuano A, Tolusso B, Pagliari G (2005). Functional, radiological and biological markers of alveolitis and infections of the lower respiratory tract in patients with systemic sclerosis. Respir Res.

[7] Mukerjee D, St George D, Coleiro B, Knight C, Denton CP, Davar J (2003). Prevalence and outcome in systemic sclerosis associated pulmonary arterial hypertension: application of a registry approach. Ann Rheum Dis.

[8] Avouac J, Airo P, Meune C, Beretta L, Dieude P, Caramaschi P (2010). Prevalence of pulmonary hypertension in systemic sclerosis in European Caucasians and metaanalysis of 5 studies. J Rheumatol.

[9] Steen VD, Medsger TA (2007). Changes in causes of death in systemic sclerosis, 1972-2002. Ann Rheum Dis.

[10] Nihtyanova SI, Schreiber BE, Ong VH, Rosenberg D, Moinzadeh P, Coghlan JG (2014). Prediction of pulmonary complications and long-term survival in systemic sclerosis. Arthritis Rheumatol.

[11] Simeon-Aznar CP, Fonollosa-Pla V, Tolosa-Vilella C, Espinosa-Garriga G, Campillo-Grau M, Ramos-Casals M (2015). Registry of the Spanish Network for Systemic Sclerosis: Survival, Prognostic Factors, and Causes of Death. Medicine (Baltimore).

[12] Maricq HR, LeRoy EC, D'Angelo WA, Medsger TA, Rodnan GP, Sharp GC (1980). Diagnostic potential of in vivo capillary microscopy in scleroderma and related disorders. Arthritis Rheum.

[13] Maricq HR, Harper FE, Khan MM, Tan EM, LeRoy EC (1983). Microvascular abnormalities as possible predictors of disease subsets in Raynaud phenomenon and early connective tissue disease. Clin Exp Rheumatol.

[14] Cutolo M, Sulli A, Pizzorni C, Accardo S (2000). Nailfold videocapillaroscopy assessment of microvascular damage in systemic sclerosis. J Rheumatol.

[15] Avouac J, Vallucci M, Smith V, Senet P, Ruiz B, Sulli A (2013). Correlations between angiogenic factors and capillaroscopic patterns in systemic sclerosis. Arthritis Res Ther.

[16] Avouac J, Lepri G, Smith V, Toniolo E, Hurabielle C, Vallet A (2017). Sequential nailfold videocapillaroscopy examinations have responsiveness to detect organ progression in systemic sclerosis. Semin Arthritis Rheum.

[17] LeRoy EC, Black C, Fleischmajer R, Jablonska S, Krieg T, Medsger TA (1988). Scleroderma (systemic sclerosis): classification, subsets and pathogenesis. J Rheumatol.

[18] van den Hoogen F, Khanna D, Fransen J, Johnson SR, Baron M, Tyndall A (2013). 2013 classification criteria for systemic sclerosis: an American College of Rheumatology/European League Against Rheumatism collaborative initiative. Ann Rheum Dis.

[19] von Elm E, Altman DG, Egger M, Pocock SJ, Gotzsche PC, Vandenbroucke JP (2007). The Strengthening the Reporting of Observational Studies in Epidemiology (STROBE) statement: guidelines for reporting observational studies. Epidemiology.

[20] Tolosa-Vilella C, Morera-Morales ML, Simeon-Aznar CP, Mari-Alfonso B, Colunga-Arguelles D, Callejas Rubio JL (2016). Digital ulcers and cutaneous subsets of systemic sclerosis: clinical, immunological, nailfold capillaroscopy, and survival differences in the Spanish RESCLE Registry. Semin Arthritis Rheum.

[21] Morales-Cardenas A, Perez-Madrid C, Arias L, Ojeda P, Mahecha MP, Rojas-Villarraga A (2016). Pulmonary involvement in systemic sclerosis. Autoimmun Rev.

[22] Coghlan JG, Denton CP, Grunig E, Bonderman D, Distler O, Khanna D (2014). Evidence-based detection of pulmonary arterial hypertension in systemic sclerosis: the DETECT study. Ann Rheum Dis.

[23] Galie N, Humbert M, Vachiery JL, Gibbs S, Lang I, Torbicki A (2016). 2015 ESC/ERS Guidelines for the diagnosis and treatment of pulmonary hypertension: The Joint Task Force for the Diagnosis and Treatment of Pulmonary Hypertension of the European Society of Cardiology (ESC) and the European Respiratory Society (ERS): Endorsed by: Association for European Paediatric and Congenital Cardiology (AEPC), International Society for Heart and Lung Transplantation (ISHLT). Eur Heart J.

[24] Fernandez-Codina A, Simeon-Aznar CP, Pinal-Fernandez I, Rodriguez-Palomares J, Pizzi MN, Hidalgo CE, et al. Cardiac involvement in systemic sclerosis: differences between clinical subsets and influence on survival. Rheumatol Int. 2017;37:75–84. 10.1007/s00296-015-3382-2. 10.1007/s00296-015-3382-2 PMC11669078 26497313

[25] Miller MR, Crapo R, Hankinson J, Brusasco V, Burgos F, Casaburi R (2005). General considerations for lung function testing. Eur Respir J.

[26] Lang RM, Badano LP, Mor-Avi V, Afilalo J, Armstrong A, Ernande L (2015). Recommendations for cardiac chamber quantification by echocardiography in adults: an update from the American Society of Echocardiography and the European Association of Cardiovascular Imaging. Eur Heart J Cardiovasc Imaging.

[27] Hofstee HM, Serne EH, Roberts C, Hesselstrand R, Scheja A, Moore TL (2012). A multicentre study on the reliability of qualitative and quantitative nail-fold videocapillaroscopy assessment. Rheumatology (Oxford).

[28] Sulli A, Secchi ME, Pizzorni C, Cutolo M (2008). Scoring the nailfold microvascular changes during the capillaroscopic analysis in systemic sclerosis patients. Ann Rheum Dis.

[29] Maricq HR (1981). Wide-field capillary microscopy. Arthritis Rheum.

[30] Donner A, Eliasziw M (1987). Sample size requirements for reliability studies. Stat Med.

[31] Walter SD, Eliasziw M, Donner A (1998). Sample size and optimal designs for reliability studies. Stat Med.

[32] Landis JR, Koch GG (1977). The measurement of observer agreement for categorical data. Biometrics.

[33] Morardet L, Avouac J, Sammour M, Baron M, Kahan A, Feydy A (2016). Late nailfold videocapillaroscopy pattern associated with hand calcinosis and acro-osteolysis in systemic sclerosis. Arthritis Care Res (Hoboken).

[34] Cutolo M, Herrick AL, Distler O, Becker MO, Beltran E, Carpentier P (2016). Nailfold videocapillaroscopic features and other clinical risk factors for digital ulcers in systemic sclerosis: a multicenter, prospective cohort study. Arthritis Rheumatol.

[35] Markusse IM, Meijs J, de Boer B, Bakker JA, Schippers HP, Schouffoer AA, et al. Predicting cardiopulmonary involvement in patients with systemic sclerosis: complementary value of nailfold videocapillaroscopy patterns and disease-specific autoantibodies. Rheumatology (Oxford). 2017;56:1081–88. 10.1093/rheumatology/kew402. 10.1093/rheumatology/kew402 27940596

[36] Caramaschi P, Canestrini S, Martinelli N, Volpe A, Pieropan S, Ferrari M (2007). Scleroderma patients nailfold videocapillaroscopic patterns are associated with disease subset and disease severity. Rheumatology (Oxford).

[37] Smith V, Decuman S, Sulli A, Bonroy C, Piettte Y, Deschepper E (2012). Do worsening scleroderma capillaroscopic patterns predict future severe organ involvement? A pilot study. Ann Rheum Dis.

[38] Silva I, Teixeira A, Oliveira J, Almeida I, Almeida R, Aguas A (2015). Endothelial dysfunction and nailfold videocapillaroscopy pattern as predictors of digital ulcers in systemic sclerosis: a cohort study and review of the literature. Clin Rev Allergy Immunol.

[39] Sebastiani M, Manfredi A, Colaci M, D'Amico R, Malagoli V, Giuggioli D (2009). Capillaroscopic skin ulcer risk index: a new prognostic tool for digital skin ulcer development in systemic sclerosis patients. Arthritis Rheum.

[40] Sebastiani M, Manfredi A, Vukatana G, Moscatelli S, Riato L, Bocci M (2012). Predictive role of capillaroscopic skin ulcer risk index in systemic sclerosis: a multicentre validation study. Ann Rheum Dis.

[41] Trombetta AC, Smith V, Pizzorni C, Meroni M, Paolino S, Cariti C (2016). Quantitative alterations of capillary diameter have a predictive value for development of the capillaroscopic systemic sclerosis pattern. J Rheumatol.

[42] Bredemeier M, Xavier RM, Capobianco KG, Restelli VG, Rohde LE, Pinotti AF (2004). Nailfold capillary microscopy can suggest pulmonary disease activity in systemic sclerosis. J Rheumatol.

[43] Castellvi I, Simeon-Aznar CP, Sarmiento M, Fortuna A, Mayos M, Geli C (2015). Association between nailfold capillaroscopy findings and pulmonary function tests in patients with systemic sclerosis. J Rheumatol.

[44] Medsger TA, Bombardieri S, Czirjak L, Scorza R, Della Rossa A, Bencivelli W (2003). Assessment of disease severity and prognosis. Clin Exp Rheumatol.

[45] Lambova SN, Muller-Ladner U (2010). Capillaroscopic pattern in systemic sclerosis—an association with dynamics of processes of angio- and vasculogenesis. Microvasc Res.

[46] Hofstee HM, Vonk Noordegraaf A, Voskuyl AE, Dijkmans BA, Postmus PE, Smulders YM (2009). Nailfold capillary density is associated with the presence and severity of pulmonary arterial hypertension in systemic sclerosis. Ann Rheum Dis.

[47] Riccieri V, Vasile M, Iannace N, Stefanantoni K, Sciarra I, Vizza CD (2013). Systemic sclerosis patients with and without pulmonary arterial hypertension: a nailfold capillaroscopy study. Rheumatology (Oxford).

[48] Corrado A, Correale M, Mansueto N, Monaco I, Carriero A, Mele A (2017). Nailfold capillaroscopic changes in patients with idiopathic pulmonary arterial hypertension and systemic sclerosis-related pulmonary arterial hypertension. Microvasc Res.

[49] Miniati I, Guiducci S, Conforti ML, Rogai V, Fiori G, Cinelli M (2009). Autologous stem cell transplantation improves microcirculation in systemic sclerosis. Ann Rheum Dis.

